# Impact of Customized and Sustained Physiotherapy in Charcot-Marie-Tooth Disease

**DOI:** 10.7759/cureus.17201

**Published:** 2021-08-15

**Authors:** Tajuddin Chitapure, Divya Jethwani, Syed Zubair Ahmed, Chinmoyee Panigrahy

**Affiliations:** 1 Assistant Professor, MGM School of Physiotherapy, Aurangabad, a constituent unit of MGMIHS, Navi Mumbai, IND; 2 Associate Professor, Department of Physiotherapy, Tilak Maharashtra Vidyapeeth Jayantrao Tilak College of Physiotherapy, Pune, IND; 3 Neurophysiotherapy, Royal College of Physiotherapy, Malegaon, IND; 4 Musculoskeletal Physiotherapy, Tilak Maharashtra Vidyapeeth Jayantrao Tilak College of Physiotherapy, Pune, IND

**Keywords:** charcot-marie-tooth disease, polyneuropathy, physiotherapy, emg-ncv, functional independence

## Abstract

Charcot-Marie-Tooth (CMT) disease is the most inherited form of peripheral neuropathy. This condition is also known as hereditary motor and sensory neuropathy (HMSN), which is a slowly progressive neuropathy affecting peripheral nerves and causes sensory loss, weakness and muscle wasting. This primarily involves distal muscles of feet, lower legs, hands and forearm. CMT is the most frequently inherited peripheral neuropathy known to affect 1 in 2500 individuals. There are four types: CMT1, CMT2, CMT3 and CMT4, depending upon the involvement of specific gene deficit, inheritance pattern, age of onset and whether the primary defect results in an abnormality of the myelin or axon of the nerve. Diagnosis of CMT is done based on physical examination, genetic testing, and electromyography (EMG) and nerve conduction velocity (NCV) test results. Symptoms of weakness progress from distal to proximal, i.e., it begins from feet and ankles. CMT is a known length dependent neuropathy which results in foot drop and later weakness of hands and forearm becomes evident. Due to the involvement of sensory nerve fibres, sensations like heat, touch, pain and most prominently vibration is also present distally. Long standing effects of CMT leads to the development of deformity and/or contractures which may involve forefoot, hindfoot, toes and/or long finger flexors. As physiotherapy is a means to maintain and/or gain maximum possible functional independence, consistency of the treatment becomes the most important factor. This case report intends to show that consistency in performing physiotherapeutic exercises helps in gaining maximum possible functional independence. This case report is a discussion of a 25-year-old male patient referred to the physiotherapy department with the polyneuropathy type symptoms with his history and investigation reports being consistent with that of Charcot-Marie-Tooth disease.

## Introduction

Charcot-Marie-Tooth (CMT) disease is the most common hereditary neuropathy, affecting approximately 10-82.3 in 100000 individuals [1]. CMT hereditary neuropathy refers to a group of disorders characterized by chronic motor and sensory polyneuropathy, also known as hereditary motor and sensory neuropathy (HMSN) [2]. Patients with CMT usually show bilateral involvement with slow progressive distal motor neuropathy of the hand and foot muscles usually beginning in the first to third decade of life and show atrophy of distal extremities like hand and feet muscles. This muscle weakness often presents with mild to moderate distal sensory loss [3]. The traditional classification of CMT (e.g., CMT1, CMT2, and DI-CMT [dominant intermediate]) was based on peripheral neuropathy type as determined by nerve conduction velocity (NCV) and mode of inheritance as determined by family history. In most studies, the treatment approach is symptomatic. Affected individuals are often evaluated and managed by a multidisciplinary team that includes neurologists, physiatrists, orthopedic surgeons, and physical and occupational therapists [4,5]. In the literature the quality of life and defining disability have been measured and compared among various groups of individuals with CMT [6,7]. In this case report, a 25-year-old male had a history of weakness in both the hand and foot muscles, which were progressive, and because of this he was unable to participate in any social and cultural activities in society and limited his daily activities. After referring to many physicians, he underwent an investigation and was diagnosed with polyneuropathy because of CMT. So with this case report, we wanted to observe if consistency in physical therapy treatment results in improvement in his functional outcome, which ultimately improves his quality of life.

This case report is about a 25-year-old male who was referred to physical therapy OPD with complaints of weakness in both the hands and feet for four years.

## Case presentation

A 25-year-old male was referred to physical therapy OPD with complaints of weakness in both the hands and feet for four years. The patient had a history of insidious onset of weakness in both the upper and lower limbs. Initially it started as a feeling of tingling and numbness in the right foot followed by the left foot. Thereafter weakness developed in both feet. He faced difficulty in performing daily activities like walking because he had to drag his feet as there was a presence of foot drop due to progressive weakness and he faced difficulty in holding slippers while walking. After a couple of months, he developed a similar type of weakness in both hands and therefore holding and grasping became difficult. As the severity was progressive, it started affecting his normal mobility components like standing balance, walking, overhead activities, holding various objects and fine skill movements. With the passage of time, he started losing sensations and because of this he developed an open wound at the ball of the great toe. He was referred to a private neurologist and underwent nerve biopsy as advised. The results showed axonopathy and but there was no evidence of inflammation. After taking physical therapy treatment for two months in some private clinics, he came to Maharashtra Railway Vikas Corporation health center physical therapy department for further management and here he underwent physical therapy treatment with regular follow-ups for three consistent months. On further assessment, family history was found to be present. Patient’s father was known to have the same presentation when he was around 30 years of age.

On observation, gross wasting of both the hands and lower leg and foot muscles was seen. The patient was using a walker to walk. Higher mental functions were intact. There was a loss of sensations over bilateral hand (volar and dorsal) and below the knee region in bilateral lower limbs. On the motor examination, the patient was found to have hypotonia in hands and lower leg muscles bilaterally.

By convention method, the deep tendon reflexes are graded as follows in Table 1.

**Table 1:** Deep Tendon Reflexes (DTR)

DTR (Deep Tendon Reflexes)		
	Right	Left
Biceps	++	++
Triceps	++	++
Supinator	+	+
Knee	+	+
Ankle	-	-

Manual muscle testing was done using Medical Research Center (MRC) Gradings and the findings are as follows in Table 2. Previous status indicates the findings during first assessment of the patient and current status denotes follow-up assessment findings taken one day before patient’s last visit.

**Table 2:** Manual Muscle Testing MRC (Medical Research Center) Grading

Muscles	Previous Status (During 1^st^ Assessment)	Current Status after 12 weeks (During a follow up assessment)
Hip and Knee Muscles	Grade 3	Grade 4
Ankle and Foot Muscles	Grade 1	Grade 2+
Shoulder Muscles	Grade 4	Grade 5
Wrist Muscles	Grade 3	Grade 5
Hand Muscles	Grade 1	Grade 2+

Range of motion was full for all joints except wrist because the presence of hypotonia made it hypermobile. Initially, there was mild tightness in hip flexors, because he uses to walk with anterior pelvic tilt, but later on successive treatment sessions, tightness was relieved and gait improvements were noticed.

Functional status of the patient is shown in Table 3.

**Table 3:** Functional Status

Hand		
Previous Status	Current Status after 12 weeks	Remark
Personal hygiene and grooming difficulties	Now able to perform	
Typing on laptop for 1-2 minutes	Now 5-10 minutes	
Holding objects in hand was difficult	Now holding objects with spherical grip is possible	Prehension grip is yet to be achieved
Gripping object with thumb opposition (e.g.: holding a bottle)	Now able to use thumb for opposition and for variable objects	
Holding book/Paper in web space was difficult	Now able to hold objects in web spaces	Interossei muscles are weak
Lower Limb		
Felt heaviness and weakness throughout the Lower Limb	Weakness present only in foot and ankle region	
1-2 minutes walking was painful	Now able to walk upto 10minutes without pain	Walks with wide base of support due to fear of fall/imbalance
Walked with hip hiking so as to be able to clear ground as there was a presence of foot drop because of loss of complete power	Now able to walk with very mild hip hiking and knee flexion, so as to clear the ground	Walks with toe out position
Lack of balance – unable to stand independently	Now independent standing is possible	
Standing from sitting on ground position required assistance	Now independent transition is possible	
Walking with lower foot orthotic support	Independent walking without orthosis is possible	

Investigations like electromyography (EMG) and nerve conduction velocity (NCV) testing were done. Results are shown in Tables 4, 5, 6.

**Table 4:** Nerve Conduction Velocity (NCV) Report Findings/Impression TL – Terminal Latency, CMAP – Compound Muscle Action Potential, MNCV – Motor Nerve Conduction Velocity, TD – Temporal Dispersion, FCR – Flexor Carpi Radialis, EDB – Extensor Digitorum Brevis, AH – Abductor Hallucis, TA – Tibialis Anterior

Case Findings	
Patient is referred for Inherited Neuropathy? Acquired Presenting Complaint: Initially patient experienced that slippers would slip off his feet while walking. After a year, patient developed an ulcer on the ball of right toe which took 10 months to heal and later on within one more year, patient started experiencing difficulty in getting up from squatting position, imbalance while walking and progressively there was presence of weakness and wasting of hand muscles.	
Comments:	
Sensory:	
Right and Left Median D2	No Response
Right Ulnar	Normal
Right Radial	Normal
Right Sural	Biopsied
Left Sural	No Response
Right and Left Superficial Peroneal	No Response
MOTOR AND F Wave	
Right Median	TL: Prolonged (demyelinating range), Distal CMAP: Low Amplitude, MNCV: Slowed in arm and forearm (demyelinating range), TD noted on stimulating at elbow and mid arm (severe)
Left Median	TL: Prolonged (demyelinating range), Distal CMAP: Low Amplitude and distally dispersed (demyelinating range), MNCV: Slowed in arm and forearm >>arm, TD noted (severe)
Right Ulnar	TL: Prolonged, CMAP: Very low amplitude and distally dispersed, MNCV: Slowed in arm and forearm (Demyelinating range), TD noted (mild)
Right Median – FCR	Normal
Right Common Peroneal – EDB	No Response
Right and Left Tibial – AH	No Response
Right and Left Tibial – Gastrocnemius	CMAP: Very Low Amplitude
Right Common Peroneal – TA	CMAP: Very Low Amplitude
Left Common Peroneal – TA	No Response
Right Femoral	TL: Mildly Prolonged, CMAP: Normal Amplitude
Left Femoral	Normal
Note: Skin Temperature: Upper Limb>32^0^C, Lower Limb > 30^0^C (throughout the test) NCV Findings as listed below: There is evidence of a widespread, multifocal peripheral neuropathy. Type: Motor>Sensory, Lower Limb + Upper Limb, Distal > Proximal, Demyelinating and Axonal Suggestive of an inherited variety e.g. CMT-X (due to paucity of sensory symptoms)	

**Table 5:** Motor Nerve Conduction Velocity (MNCV) Report NCS – nerve conduction study, FCR – Flexor Carpi Radialis

Motor NCS										
Nerve / Sites	Amp. 2-4mV	Lat. Ms	Amp.1-2 mV	Dur. %	Area %	Dur. ms	Amp.2-4%	Dist. Cm	Vel. m/s	Rec. Site
Right Median										
Wrist	2.4	5.15	1.9	100	100	6.50	100	6		APB
Elbow	1.0	13.95	0.8	197	81.1	12.80	41.5	22	25.0	
Mid Arm	1.1	16.55	0.8	179	9.6	11.65	45	7	26.9	
Left Median										
Wrist	3.2	5.15	2.4	100	100	8.35	100	6		APB
Elbow	2.1	15.30	1.7	150	107	12.55	64.7	22.5	22.2	
Mid Arm	2.0	16.75	1.6	160	103	13.40	63.2	7	48.3	
Right Ulnar										
Wrist	1.4	4.55	0.8	100	100	7.65	100	6		ADM
Mid Elbow	1.4	13.65	0.8	182	152	13.90	99.3	22	24.2	
Mid Arm	1.4	16.80	0.8	125	81.3	7.90	54.2	8	32.0	
Left Ulnar										
Wrist	2.0	3.85	1.5	100	100	6.30	100	6		ADM
Mid Elbow	1.2	12.85	0.9	121	82.6	7.60	57.6	23	25.6	
Mid Arm	1.1	15.35	0.8	125	81.3	7.90	54.2	8	32.0	
Right Common Peroneal - TA										
Fib Head	0.6	8.30	0.4	100	100	13.00	100			
Left Tibial Malleolus – Gastroc										
Knee	0.5	6.60	0.4	100	100	15.75	100			Gastroc
2	0.5	6.50	0.5	106	116	16.75	106			Gastroc
Right Tibial Malloelus – Gastroc										
Knee	0.3	6.80	0.3	100	100	14.95	100			Gastroc
2										Gastroc
Right Femoral										
Groin	8.6	4.30	5.7	100	100	14.65	100			Rectus Femoris
Thigh	8.1	4.20	5.5	100	94.1	14.65	93.5			
Left Femoral										
Groin	12.1	2.50	7.5	100	100	14.65	100			Rectus Femoris
Right Median – FCR										
Elbow	14.0	1.75	9.2	100	100	8.95	100			FCR
R Common Peroneal : No Response L Tibial Malleolus: No Response R Tibial Malleolus: No Response L Common Peroneal TA: No Response F Wave: L Ulnar – ADM : Min F Lat = 32.15ms										
Sensory NCS										
R Sup Peroneal: No Response L Sural: No Response L Sup Peroneal: No Response R Median: No Response R Ulnar: No Response L Median: No Response										

**Table 6:** Electromyography (EMG) Report MUPs – Motor Unit Potentials

NEEDLE ELECTROMYOGRAPHY				
Muscle Name	Spontaneous Activity	Voluntary Activity	Interference Pattern	Remarks
Right Vastus Medialis (Femoral Nerve L2,3,4)	NIL	Large wide MUPs+Large Polyphasics	Moderately Reduced	Chronic Partial Denervation
Right Tibialis Anterior (Deep Peroneal Nerve L4,5)	Fibs 2+	Fast Firing Polyphasics	Severely Reduced	Active and Chronic Partial Denervation
Right First Dorsal Interosseous (Ulnar Nerve: C8,T1)	Fibs 3+	Large Wide MUPs	Severely Reduced	Active and Chronic Partial Denervation
Right Biceps (Musculocutaneous Nerve C5,6)	NIL	Normal	Full	Normal
Left Vastus Medialis (Femoral Nerve L2,3,4)	NIL	Large Wide MUPs+Large Polyphasics	Mildly Reduced	Chronic Partial Denervation
Left Exrtensor Indices (Radial Posterior Interosseous Nerve C7,8)	NIL	Normal	Full	Normal

The patient was diagnosed with polyneuropathy because of Charcot-Marie-Tooth disease (CMT) based on the EMG+NCV study’s findings as well as history and familial presentation along with the progression of the symptoms.

Diagnostic challenges

Hereditary neuropathies are divided into four major subcategories: hereditary motor and sensory neuropathy, hereditary sensory neuropathy, hereditary motor neuropathy, and hereditary sensory and autonomic neuropathy. The most common type is Charcot-Marie-Tooth disease, one of the hereditary motor and sensory neuropathies. Symptoms of the hereditary neuropathies vary according to the type and may include sensory symptoms such as numbness, tingling, and pain in the feet and hands, or motor symptoms such as weakness and loss of muscle bulk, particularly in the lower leg and feet muscles. Certain types of hereditary neuropathies can affect the autonomic nerves, resulting in impaired sweating, postural hypotension, or insensitivity to pain. Some people may have foot deformities such as high arches and hammer toes, thin calf muscles (having the appearance of an inverted champagne glass) or scoliosis (curvature of the spine). The symptoms of hereditary neuropathies may be apparent at birth or appear in the middle or late life. They can vary among different family members, with some family members being more severely affected than others. The hereditary neuropathies can be diagnosed by blood tests for genetic testing, nerve conduction studies, and nerve biopsies.

Diagnosis was made based on EMG+NCV study reports and familial predisposition. Also on examination, diabetes was ruled out to rule out for diabetic neuropathy. The patient was suggested to undergo nerve biopsy, but because of financial constraints patient denied opting for it.

Intervention

Electrical Stimulation 

Interrupted galvanic current used to target intrinsic muscles of the hand and foot muscle with 90-90-90 set of contortions given. Along with the facilitation techniques were used to enhance the outcome of muscle contraction, which is achieved through electrical stimulation. This was continued for three weeks.

Strengthening Exercises

For lower limb and pelvis muscles was done as it was grade 3 in the initial. With the help of De Lorme’s; progressive resistance exercise (PRE) program based on 10 repetitions maximum (10RM) where the patient begins sets of training by performing the first set of 10 at 50% 10RM, the second at 75% 10RM and the third (final) at 100% of the 10RM. This was started at the end of two weeks to six weeks of treatment duration.

**Figure 1:**
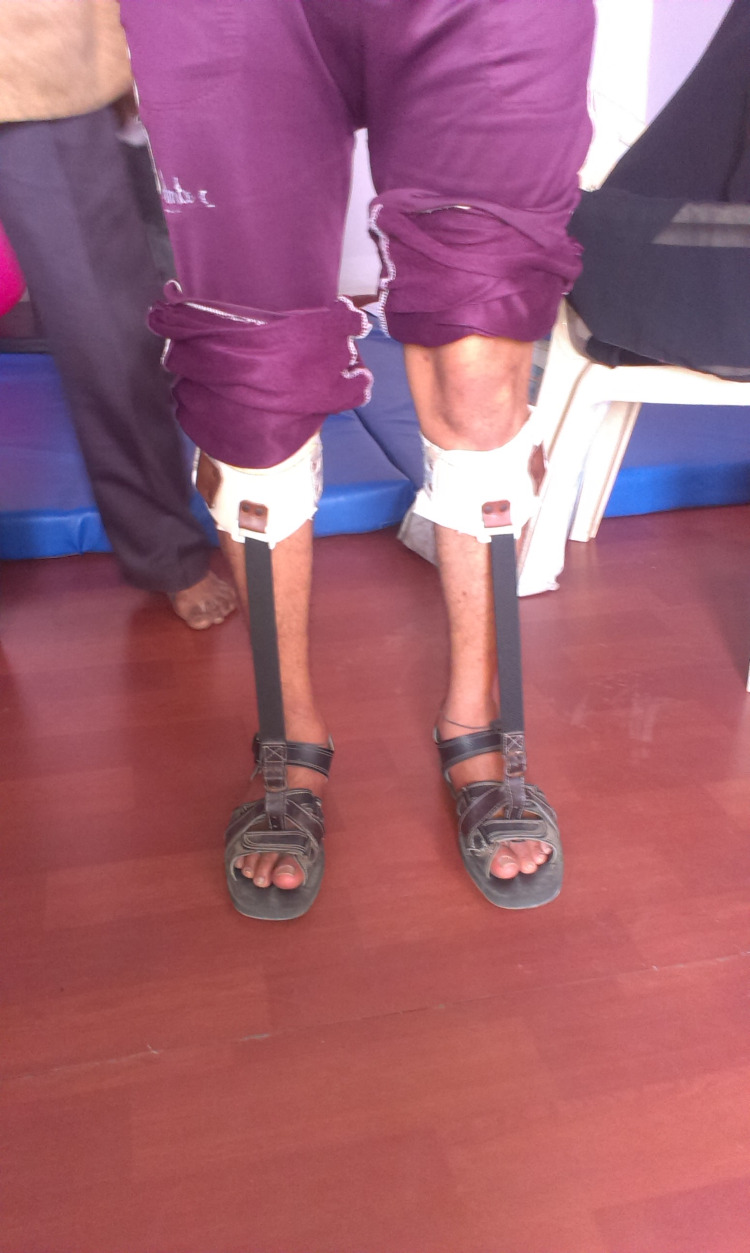
With Orthosis

At the end of six weeks core and abdominal strengthening exercises were also carried out to enhance the lower extremity muscle function. There are dearth of literature suggests that core stability is an important component of nearly every gross motor activity. The reason behind core strengthening proving effective in development of extremity strength can be justified by overflow irradiation concept which says when a resistance is applied; it causes irradiation or overflow from stronger patterns to weaker patterns or from stronger muscles to weaker muscles within a pattern of movement. Also the role of core is considered important in upper and lower proximities strengthening as the maintenance of position in which strengthening is performed have activation and recruitment of core muscles in order to maintain truncal stability. 

More of functional task in the form of gripping activities were done using different objects of different shapes. Stretching of tight muscles was done and once all these things were achieved, patient was mad to undergo gait training first in parallel bar and later out of the parallel bar. We used the plantar flexed assist orthosis to improve his gait function and facilitated the dorsiflexor muscles. Once gait was improved markedly, patient was put under endurance training via static cycling.

At the end of eight weeks balance exercises were started with dynamic sitting balance training followed by static balance training in standing followed by dynamic balance training in standing and later on balance exercises on dynamic surfaces like physio ball and balance board were given. 

Also endurance training was done incorporated in form of breathing exercises, static cycling and walking with normal speed and in normal pattern.

Limitations

As this disease is progressive, sustained exercises must be implemented for prolonged functional independence and also care must be taken to make sure exercises focus on maintaining muscle properties so that neural status affection would cause a slow deterioration in activities of daily living (ADL) performance.

## Discussion

Although CMT is an incurable disease, quality of life for patients with CMT can be improved by symptomatic treatments like physical therapy, surgery wherever indicated, analgesics and other pain management strategies, etc [8]. To establish a program specific for each patient, the management in physical medicine and rehabilitation of patients with Charcot-Marie-Tooth disease must be early after a clinical assessment of joint, muscles and sensory status [9], as CMT is the most common hereditary sensorimotor neuropathy that has a slow onset. CMT is usually first present in childhood. It starts distally and from the lower limbs it progresses to more proximal muscles. Rehabilitation plays a major role in the treatment of patients with CMT due to the lack of curative medical treatments and the problematic outcomes of surgical intervention. Important aspects of rehabilitation are approaches like aerobic, stretching and strengthening exercises. Rehabilitative approach is incomplete without orthotics. Evidence shows that strength and general fitness are greatly improved by exercises. Range of motion is effectively maintained by customized stretching protocol along with orthotic devices, which are the mainstay of maintaining mobility and ambulation and upper extremity function [10]. In this patient also sustained physical therapy showed marked improvement in terms of strength, range of motion, balance and gait and overall functional independence. This case report aims to show the significance of sustained physical therapy and an effective physical therapy protocol to yield functional independence in Charcot-Marie-Tooth disease. Evidence agrees on the improvement in timing of execution of ADLs and overall functional independence [11]. Appropriate inclusion of aerobic exercises improving endurance maintains the symptoms from worsening up in progressive disorders [12].

## Conclusions

This case report concludes that early physical therapy with customized protocol and sustained approach improves overall functional independence of the patient with polyneuropathy due to Charcot-Marie-Tooth disease. Appropriate workplace modification and appropriate orthotic support add up to the quality of life of these patients.
